# A mobile app for improving the compliance with remote management of patients with cardiac implantable devices: a multicenter evaluation in clinical practice

**DOI:** 10.1007/s10840-022-01207-y

**Published:** 2022-04-12

**Authors:** Carlo Lavalle, Michele Magnocavallo, Alessia Bernardini, Giampaolo Vetta, Valter Bianchi, Agostino Mattera, Marco Valerio Mariani, Ernesto Ammendola, Giuseppe Busacca, Agostino Piro, Carmen Adduci, Leonardo Calò, Luca Panchetti, Stefano Viani, Antonio Rapacciuolo, Giampaolo Sanna, Giulio Molon, Fabio Quartieri, Rita Di Rosa, Monica Campari, Sergio Valsecchi, Antonio D’Onofrio

**Affiliations:** 1 Department of Cardiovascular, Respiratory, Nephrology, Anaesthesiology and Geriatric Sciences, Sapienza University of Rome, Viale del Policlinico, Rome, 00161 Italy; 2 Unità Operativa di Elettrofisiologia, Studio e Terapia delle Aritmie, Monaldi Hospital, Naples, Italy; 3 S. Anna e S. Sebastiano Hospital, Caserta, Italy; 4 Second University of Naples, A.O. Monaldi, Naples, Italy; 5 Ospedale E. Muscatello, Augusta, Italy; 6 Division of Cardiology, Department of Clinical and Molecular Medicine, Sapienza University of Rome, St. Andrea Hospital, Rome, Italy; 7 Policlinico Casilino, Rome, Italy; 8 Scuola Superiore Sant’Anna and Fondazione Toscana Gabriele Monasterio, Pisa, Italy; 9 University Hospital of Pisa, Pisa, Italy; 10 Policlinico Federico II, Naples, Italy; 11 P.O. San Martino, Oristano, Italy; 12 Sacro Cuore-Don Calabria Hospital, Verona, Italy; 13 Arrhythmology Centre, Arcispedale S. Maria Nuova, Reggio Emilia, Italy; 14 Ospedale Santa Maria Goretti, Latina, Italy; 15 Boston Scientific, Milan, Italy

**Keywords:** Cardiac implantable electronic device, Remote monitoring, Mobile app, Follow-up

## Abstract

**Background:**

The remote device management (RM) is recommended for patients with cardiac implantable electronic devices (CIEDs). RM underutilization is frequently driven by the lack of correct system activation. The MyLATITUDE Patient App (Boston Scientific) has been developed to encourage patient compliance with RM by providing information on communicator setup, troubleshooting, and connection status of the communicator.

**Methods:**

At 14 centers, patients with CIEDs were invited to download and install the App on a mobile device. After 3 months, patients were asked to complete an *ad hoc* questionnaire to evaluate their experience.

**Results:**

The App was proposed to 242 consecutive patients: 81 before RM activation, and 161 during follow-up. The App was successfully installed by 177 (73%) patients. The time required for activation of the communicator and the need for additional support were similar between patients who followed the indications provided by the App and those who underwent standard in-clinic training. During follow-up, notifications of lack of connection were received by 20 (11%) patients and missed transmission by 22 (12%). The median time from notification to resolution was 2 days. After 3 months, 175 (99%) communicators of the 177 patients who installed the App were in “Monitored” status versus 113 (94%) of 120 patients without the App installed (*p*=0.033). The use of the app made 84% of patients feel reassured.

**Conclusions:**

The App was well accepted by CIED patients and offered support for communicator management and installation. Its use enabled patients to remain connected with greater continuity during follow-up.

## Introduction

According to current guidelines, the use of remote management (RM) is recommended for patients with cardiac implantable electronic devices (CIEDs) [1, 2]. Several trials have compared in-person evaluations and RM for the follow-up care of CIED patients and have explored the ability of RM to detect problems early, thereby improving patient outcomes [3–6]. The advent of wireless RM and novel diagnostic features that enable devices to monitor their own functions, record arrhythmias and physiological parameters, and automatically communicate this information to healthcare providers without the active participation of the patient has been critical to achieving the follow-up goals of patient adherence to structured follow-up protocols and to improving the workflow efficiency of device clinics [2, 7, 8]. Successful transmission of RM data by the patient to the healthcare provider relies on the enrollment of the patient in the specific RM system and the subsequent activation and maintenance of RM by the patient. Enrollment in RM has been shown to depend largely upon the local practice of the institution. However, RM activation and transmission by the patient depend upon patient factors [9]. The continuity of monitoring is crucial. Indeed, patients who consistently transmit data by means of RM are at substantially lower risk of death and readmission to hospital [10].

The MyLATITUDE™ Patient App (Boston Scientific) has been developed to encourage patients’ compliance with RM by providing them with information on communicator setup and troubleshooting, connection status of the communicator, scheduled transmissions, and status of the battery of the implanted device.

In the present study, we evaluated the first experience of the adoption of the MyLATITUDE™ Patient App in clinical practice.

## Methods

From May to July 2021, at 14 Italian arrhythmia centers, patients with a compatible Boston Scientific CIED with RM capabilities were invited to download and install the App on a mobile device.

The use of the App was proposed to all consecutive patients after CIED implantation or, during a scheduled follow-up visit, to consecutive patients already enrolled and monitored at home through the LATITUDE™ platform. The study design was approved by the Institutional Committee on Human Research of each center and informed consent was obtained from all patients. According to individual patient preference, patients were assigned to the App group or the control group.

In the App group, patients received instructions on how to download the App from the App Store of their own mobile device. The LATITUDE™ communicator was given to all patients before discharge or directly delivered to their homes, according to the standard practice of the center. Patients were invited to follow the indications provided by the App in order to activate the communicator and perform the first transmission. If they had any difficulties, patients could contact the center or the Boston Scientific customer support service. After activation, patients were instructed to follow the indications provided by the App in order to solve possible connection problems in the event of notification of “Not Monitored” status or missed scheduled remote transmission. The group of patients already monitored at home through the LATITUDE™ platform and invited to use the App were included in the analysis of the compliance with remote monitoring. An additional group of 60 consecutive patients served as control group and received standard in-clinic training on the activation and use of the communicator, as well as the patients who did not install the App. In November, the monitoring status of all patients with a LATITUDE™ communicator was checked. Patients in “Monitored” status were considered compliant with remote monitoring at medium-term follow-up. All patients were asked to reply to an *ad hoc* questionnaire to collect anonymous data and evaluate their experience. All data were collected under local standard-of-care conditions of use. The operators at the centers were also asked to complete a questionnaire to provide their feedback on the adoption of the App.

### MyLATITUDE™ Patient App

**Fig. 1:**
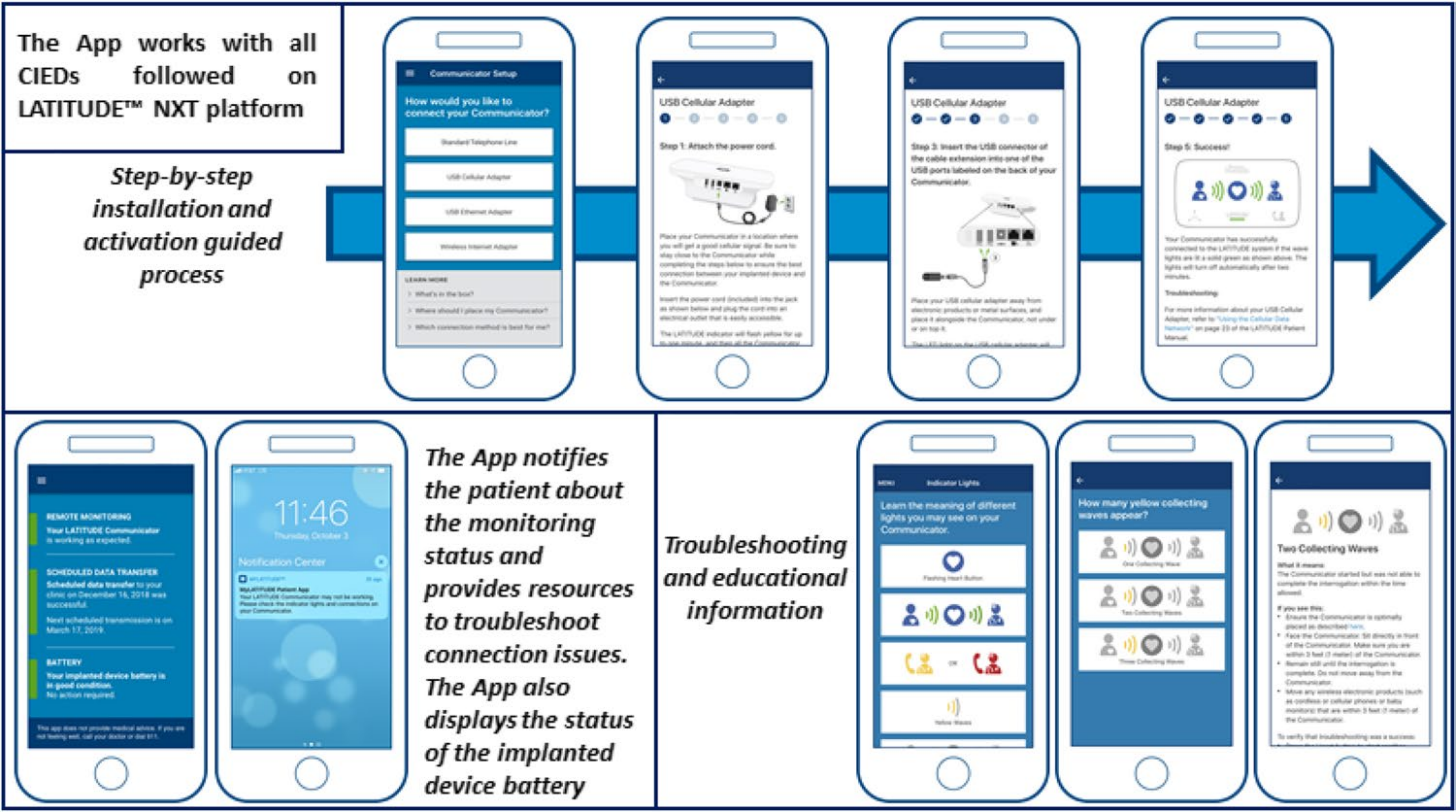
The MyLATITUDE™ Patient App enables patients to independently obtain instruction and information on their LATITUDE™ NXT Communicator and implanted device

The App enables patients to independently obtain instructions and information on their communicator and CIED. It works with all Boston Scientific CIEDs that are monitored on the LATITUDE™ NXT platform and can be used by patients and/or their caregivers (Fig. 1). The App is available for iOS and Android mobile phones; it does not connect directly to the patient’s device but receives the data from the server. The App provides easy-to-follow steps that enable patients to set up their communicator. If the patient’s communicator is not in a “Monitored” state, the App notifies the patient and provides resources to troubleshoot the problem, in order to encourage patient compliance with RM and to reduce the center’s burden of managing “Not Monitored” patients. Moreover, the App informs patients of the status of scheduled remote transmission and of the CIED battery.

### Statistical analysis

Descriptive statistics are reported as means ± standard deviation for normally distributed continuous variables or medians with 25th to 75th percentiles in the case of skewed distribution. Categorical data were expressed as percentages. Differences between mean data were compared by means of a *t*-test for Gaussian variables and Mann-Whitney non-parametric test for non-Gaussian variables. Differences in proportions were compared by means of a chi-square analysis. A *p*-value <0.05 was considered significant for all tests. All statistical analyses were performed by means of *R*: a language and environment for statistical computing (R Foundation for Statistical Computing, Vienna, Austria).

## Results

### Participating centers and device management service

The MyLATITUDE™ Patient App was proposed to patients with a compatible CIED at 14 Italian arrhythmia centers. At 11 (79%) centers, the professionals routinely involved in remote device management were nurses and physicians, in accordance with a “Primary Nursing” model. In the remaining 3 (21%) centers, they were physicians alone. The communicator was delivered by nurses at 11 (79%) centers and by physicians at 3 (21%) centers. At 6 (43%) centers, home delivery of the communicator was allowed if preferred by the patient. The professionals involved in training patients in the activation and use of the communicator were nurses at 9 (64%) centers and physicians at 5 (36%) centers.

### Acceptance and installation of the App

The App was proposed to 242 patients: 81 consecutive patients immediately after CIED implantation and 161 patients who were already being monitored at home through the LATITUDE™ platform and who were consecutively enrolled at the time of an in-clinic visit. The App was successfully installed by 177 (73%) patients; 49 (20%) declined, 12 (5%) did not have a suitable mobile phone, and 4 (2%) experienced technical problems that precluded LATITUDE™ communicator activation (lack of landline and mobile telephone coverage). Baseline parameters of the study population are reported in Table 1. Patients who installed the App were younger and had a higher level of education. The App was more frequently accepted by patients already using RM.

**Table 1:** Baseline parameters of the patients invited to use the App and comparison between those who installed it and those who did not

	Patients invited to use the App (242)	App installed (177)	App not installed (65)	*p*
Male gender, i (%)	190 (79)	139 (79)	51 (78)	0.991
Age:				
≤50 years, *n* (%)	79 (33)	67 (38)	12 (18)	0.004
51–70 years, *n* (%)	94 (39)	70 (39)	24 (37)	0.710
>70 years, *n* (%)	69 (28)	40 (23)	29 (45)	<0.001
Secondary education or higher, *n* (%)	140 (58)	115 (65)	25 (39)	<0.001
Caregiver support, *n* (%)	60 (25)	39 (22)	21 (32)	0.101
New RM activation, *n* (%)	81 (33)	48 (27)	33 (51)	<0.001
Device				
Pacemaker	44 (18)	24 (13)	20 (31)	0.002
ICD	178 (74)	141 (80)	37 (57)	<0.001
CRT	20 (8)	12 (7)	8 (12)	0.166

*RM*, remote device management; *ICD*, implantable cardioverter defibrillator; *CRT*, cardiac resynchronization therapy

The App was installed by 127 (72%) patients, by 39 (22%) caregivers, and by patient and caregiver in 11 (6%) cases. Installation problems that did not preclude final activation were reported by 15 (8%) patients. In 12 cases, troubleshooting required telephone support.

### Installation and activation of the communicator

Forty-eight patients successfully followed the indications provided by the App on how to activate the communicator and perform the first transmission. Standard post-implantation in-clinic training was received by the 33 new RM activation patients who did not install the App and by another 60 consecutive patients. Of these latter, one (2%) patient experienced technical problems that precluded communicator activation. The duration of in-clinic training was < 30 min for all patients. The App-guided installation group and the control groups (standard in-clinic training group and App-decliners) were similar in terms of the time required for activation and the need for additional support (Table 2). Patient survey questions on the installation and activation procedure in the groups are reported in Table 2. The level of satisfaction with the information received from the App during the installation procedure was comparable to that provided by in-person training.

**Table 2:** Details of the installation and activation procedure, and survey questions on the patient experience. Comparison between patients who used the App to install and activate the communicator and those who underwent standard in-clinic training (directly or after declining to use the App)

	Standard in-clinic training (59)	App-guided installation (48)	App-decliners (33)
Need to contact the center to carry out activation, *n* (%)	1 (2)	3 (6)	3 (9)
Need to contact the technical support service to carry out activation, *n* (%)	0 (0)	1 (2)	1 (3)
Time needed to complete the activation process			
< 30 min	54 (92)	41 (85)	31 (94)
30–60 min	4 (7)	6 (13)	0 (0)
> 60 min	1 (2)	1 (2)	2 (6)
Connection problems during activation, *n* (%)	2 (3)	1 (2)	4 (12)
The information received (during in-person training or through the App) was clear and adequate for installation of the communicator	58 (98)	43 (90)	31 (94)
Would you have preferred additional information (or in-person training for App users)?	9 (15)	14 (29)	2 (6)

### Use of the App during follow-up

Of the 177 patients who installed the App, 20 (11%) received notifications of lack of connection during follow-up and 22 (12%) information of missed scheduled transmissions. The median time between notification of “Not monitored” status and resolution was 2 days (<1 week in 90%). After 3 months, 175 (99%) of these 177 communicators were in “Monitored” status. Of the 120 patients without the App installed, 113 (94%) were in “Monitored” status at the end of the observation (*p*=0.033). In particular, 48 (100%) of 48 patients who installed the App immediately after CIED implantation and 54 (92%) of 59 patients of the standard in-clinic training group (excluding App-decliners) were in “Monitored” status at the end of the observation (*p*=0.063).

The continuous monitoring of the connection status during follow-up made patients feel reassured (Table 3). Survey questions on operator experience with the App are reported in Table 4. Most operators judged the App to be an effective and efficient tool. Nonetheless, in the opinion of those operators who were not inclined to extensively suggest the use of the App, the best targets are younger and more highly educated patients or those who can be supported by a caregiver.

**Table 3:** Patient survey questions on the experience with the App during follow-up

Use of the App during follow-up (177)	
The information and notifications received from the App are clear and adequate for management of the communicator	166 (94)
The App is useful in order to verify the monitoring status	
Strongly agree	99 (56)
Agree	63 (36)
Disagree	13 (7)
Strongly disagree	2 (1)
Having information on the monitoring status is reassuring	149 (84)

**Table 4:** Survey questions on operator experience with the App (14 respondents)

Operator experience with the App	
The App is an effective tool for the installation and activation of the communicator in comparison with standard in-clinic training	
More effective	6 (43)
Equally effective	7 (50)
Less effective	1* (7)
The App improves the efficiency of the center regarding installation and activation of the communicator	
Agree	7 (50)
Neutral	5 (36)
Disagree	2** (14)
The App is an effective tool for verifying the monitoring status	
Yes	14 (100)
The App improves the efficiency of the center in terms of patient management during follow-up	
Yes	14 (100)
Patients suited to using the App *	
All patients	3 (21)
Younger patients	10 (48)
Higher education grade and familiar with technology	6 (43)
With caregiver support	5 (36)
*: multiple answers	
Implanted device suited to monitoring with the App	
All types (pacemaker, ICD, CRT)	14 (100)

**Operator’s comments:** *: “the center's patients are mostly elderly and have greater difficulty in using the App”; **: “for elderly patients without caregiver support, it is more time-consuming”

## Discussion

In the present study, we described the introduction into clinical practice of a mobile App designed to help CIED patients install and use an RM communicator and to provide notifications on the connection status of the device.

The MyLATITUDE™ Patient App was accepted by a high proportion of CIED patients. Moreover, installation of the App was completed successfully in most patients. The App was more frequently installed by younger and more highly educated patients and especially by those who were already enrolled and monitored at home through the RM platform. Indeed, the implantation of a CIED constitutes a major change for the patient, who might need some time before trying to learn about RM and additional tools. Although in most cases the App was directly installed by the patients themselves, when a caregiver was available, it was installed by this latter. Indeed, the support of a caregiver can help to overcome the reluctance of older and less tech-savvy patients.

Patients who installed the LATITUDE™ communicator under the guidance of the App did so just as successfully as those who had undergone standard post-implantation training of about 30 min in hospital; they had no additional problems, and installation time was similar. Moreover, the level of satisfaction with the App-guided installation was high, and, although approximately 30% of patients responded that they would have preferred in-person training, it is noteworthy that 20% of patients in the control group would have preferred more extensive training. The use of the App to activate the communicators is mainly intended as a potential advantage for the centers, which save the time usually devoted to in-person training and hopefully receive fewer requests for support, as patients can troubleshoot by using the App.

In patients who enabled the App, we recorded notifications of missed transmissions or lack of connection during follow-up. After notification, problems of connection were promptly solved by the patients. Consequently, these patients were able to remain connected with greater continuity than a similar group of patients who did not use the App, with a very high proportion of communicators regularly transmitting data at 3 months (99% versus 94%, *p*=0.033). This finding was obtained in the overall group of patients who installed the App and confirmed, although with no statistical significance (100% versus 92%, *p*=0.063), comparing patients who installed the App immediately after CIED implantation and patients of the standard in-clinic training group. This finding may have clinical implications. Indeed, previous analyses revealed not only better survival of patients enrolled in RM programs [11], but also an association between RM and a lower risk of mortality and re-hospitalization in the real world. Specifically, one analysis showed that the adjusted hazard of mortality up to 3 years was significantly lower among patients on active RM at 90 days (*HR* 0.80, *95% CI* 0.76–0.84) [10].

Barriers to the widespread use of RM can arise at the time of program implementation in the center and also owing to difficulties in activating RM and keeping patients monitored. While the implementation depends largely on the local practice environment and the institution, RM activation and transmission are more dependent on patient factors [9]. The adoption of the MyLATITUDE™ Patient App made patients feel reassured, seemed effective in helping them stay compliant with RM, and may possibly promote their engagement in their own healthcare.

### Improving effectiveness and efficiency of remote management of CIEDs

The remote management of CIEDs has been proposed as a strategy to improve the efficiency of device follow-up by replacing in-office device follow-up visits with remote transmissions [12–14]. Moreover, RM enables continuous assessment of device-related and clinical parameters, allowing early detection of device malfunction [15–17] or clinically relevant events [18–20], thereby potentially improving clinical outcomes [11, 21, 22]. Thus, current guidelines suggest the routine use of RM [1, 2], a recommendation that is even stronger in the era of the coronavirus disease 19 (COVID-19) pandemic, in order to maintain a high degree of safety and limit face-to-face interactions [23–26]. According to a survey by the European Heart Rhythm Association, the main barriers to the adoption of remote monitoring are the lack of reimbursement and the increased workload [27]. Although the COVID-19 pandemic has caused an acceleration in the use of RM of CIEDs, and also the establishment of reimbursement codes in some European regions, the affordability of the model for the centers that decide to implement an RM service remains a critical issue [28, 29]. The adoption of tools like the MyLATITUDE™ Patient App may be an efficient way to ensure continuity of patient monitoring while maintaining under control the workload at the centers. This could be achieved by reducing the time spent on training and support for technical troubleshooting and on managing missed transmissions, which are known to impair the clinical efficiency of RM [30].

We recently implemented direct home delivery of the communicator and remote training of patients during the COVID-19 lockdown period in Italy [31]. This strategy proved feasible, enabling the RM of all previously unmonitored CIED patients, without requiring access to the hospital [32]. The MyLATITUDE™ Patient App could be effectively adopted to support that strategy, by enabling patients to activate their communicator even without traditional in-person training. As proposed by many RM programs, after CIED implantation, the implementation of RM could be deferred [2]. When patients are at home and have had the time to recover, accept the implanted device, and process the first CIED-related information, they may receive the communicator and proceed with installation by themselves, with the aid of the mobile App.

### Limitations

Our findings have potential limitations. This was an observational non-randomized study. Some analyses were performed with patients self-selecting whether to be in the intervention or control group. Thus, a bias could affect our findings. As the project was limited to a single RM platform, our results may not be applicable to other systems. Moreover, we cannot exclude possible differences in the implementation of the initiative among centers, with an impact on the degree of success.

## Conclusion

The MyLATITUDE™ Patient App proved to be well accepted by CIED patients. It offered support for communicator installation and management and reassured patients. Its use allowed patients to remain connected with greater continuity during follow-up.

## Abbreviations

CIED: Cardiac implantable electronic device
COVID-19: Coronavirus disease 19
RM: Remote management
